# A Sketch of Psychiatry in the Southern States

**Published:** 1898-01

**Authors:** T. O. Powell

**Affiliations:** Milledgeville, Ga., Medical Superintendent State Lunatic Asylum


### A SKETCH OF PSYCHIATRY IN THE SOUTHERN STATES.*

By T. O. POWELL, M.D., MILLEDGEVILLE, GA.,

Medical Superintendent State Lunatic Asylum.

In suggesting a subject for this address, your committee stated that "due credit has never been given to the movement which brought about the erection of the buildings for the insane at the South, and it seems desirable that some special reference be made to the work done by pioneers in that field."

The theme thus briefly indicated is most inviting, and the subject, as must be confessed, has been too long neglected. But not of my own volition, nor without hesitation, would I assume the rôle of historian of the rise and progress of a vast system of charities in the fifteen commonwealths of the South. As this meeting is held in the Southern metropolis, especially renowed for her fostering care of the insane, the occasion seems to demand that the

*Abstract of address before the American Medico-Psychological Association at Baltimore, May 11, 1897.

subject proposed by the committee be taken up and dealt with comprehensively, and, so far as possible, according to its high merits. But it is scarcely to be expected that, within the narrow compass of an address such as this, full justice can be done a subject at once so vast in extent, so fertile in material, and so full of tender memories of self-sacrificing men and women long since gone to their reward, leaving us heirs of amplest inheritance.

In attempting to comply with the request of your committee, I have been sadly conscious of the deficiencies and shortcomings of my essay. And while I have endeavored to pay due tribute to many honored heroes, I have failed to trace even in my own State to the fountain source the earliest hospital movement. Miss Dix, more than any other, was the leading spirit in many Southern States, as she was elsewhere in America and abroad. But before Miss Dix, the good work had begun in Virginia, Maryland, Kentucky, South Carolina and Georgia.

For the *subject-matter* of my address I am indebted to many sources. In some instances I have been fortunate enough to discover living descendants, relatives or friends of the men whose achievements I have attempted to transcribe. They have aided me with brief biographies. While I cannot name here all who have thus placed me under obligation, I may be permitted to single out from their number as especially worthy of our gratitude, Miss Mary Galt of Virginia. Many asylum physicians, both active and retired, have given me freely of their knowledge of their predecessors, but with the prevailing modesty of their class they prefer to be nameless here.

It is my desire to pay tribute to the memories of a group of men whose lives were devoted with singleness of purpose to our calling, but whose remoteness from each other, and from the great centers, tended to dim, if not to obscure, their light and their true worth. Many of them were men of recognized character, ability and experience, but they were averse to taking prominent part in either local or national gatherings.

*Primus inter pares* was Dr. John M. Galt, the younger, of Virginia. He was generally recognized as the most notable writer on neurology and insanity among the earlier generation of Southern alienists. Searching the literature on the treatment of madness, he compiled a work on that subject now almost forgotten. Dr. Galt, Dr. Stribling and Dr. Richard S. Steuart were conspicuous examples of the earlier alienists who lived for the good of the insane and the advancement of psychiatry.

In passing, it is here worthy of mention that the idea of organizing this Association was first conceived at Staunton, Virginia, by Dr. Stribling of Virginia, and Dr. Woodward of Massachusetts. The project of such an organization having met with the hearty approval and co-operation of Drs. Kirkbride and Awl, the famous original thirteen were first convened in Philadelphia, October 16, 1844. The history of the subsequent development of the Association has so recently been reviewed as to be fresh in the minds of you all.

Of a later period, our most distinguished writers were Dr. Peter Bryce of Tuscaloosa, and Dr. John H. Callender of Nashville.

No record of Southern lunacy administration would be complete without paying homage to the memory of Miss Dorothea L. Dix, the most deserving of sainthood of all the heroes and heroines of this marvelous nineteenth century, for whom we find no human parallel save only John Howard.

Since 1845, when she went on her self-appointed mission as far south as Louisiana, her influence has been felt in every Southern State. In 1849 the site of the hospital at Raleigh, N. C., was named in her honor, and only last year a fund she had long ago collected was instrumental in enabling the South Carolina Hospital to acquire an adjoining estate, a step that in importance is second only to the foundation of the institution.

Upon finding by her own laborious inquiries that an asylum was needed in any community, she marshalled her facts so pathetically and forcibly that they appealed to the most indifferent. In presenting to State Legislatures her various memorials, she sought out the men of greatest ability and influence to champion the cause she had made her own. Having once won the leaders, as well by her womanly kindness and sympathy as by her arguments, she left the cause in their hands. Though sometimes at first unsuccessful, she was indefatigable in fulfilling her holy mission, and in the end always won.

To her personal influence is due the establishment or development of insane hospitals in ten Southern States—Maryland, North Carolina, South Carolina, Georgia, Alabama, Mississippi, Louisiana, Tennessee, Kentucky and Missouri, as well as in the District of Columbia at Washington.

Thus the latter-day saint journeyed through our Southland starting the work here, stimulating it there, aiding by criticism, encouragement and advice where each was needed. The result of such victories of peace as she achieved shall throughout all time be felt in the remotest hamlets of our section. We know that she lies buried in the beautiful cemetery at Mount Auburn, near Boston, but if we seek her monument, we must visit the noble edifices for the insane in every State.

The first asylum exclusively for the insane in the United States was established at Williamsburg, Va., in 1769, and with that single exception there was no provision made for the insane, especially in the South, for many years. In making this statement I am not unmindful of the existence of a separate ward for the insane in the Pennsylvania Hospital at Philadelphia, dating from 1751. There has been brought to my attention by my friend, Dr. Babcock of South Carolina, some facts connected with an effort made to provide for the insane in that State which indicate the establishment in Charleston in 1762 of a madhouse, as it was called, but of this institution we have no satisfactory history, and must of course assign to the Virginia Hospital the position which I have already given it.

In 1797, the year of the incorporation of Baltimore Town as a city, some of its influential citizens, led by Mr. Jeremiah Yellot, established a hospital for the relief of indigent sick persons and for the reception and care of the insane. This was first called "The Public Hospital," then "The Maryland Hospital," and in 1838 became, by resolution of the General Assembly, "The Maryland Hospital for the Insane."

In passing, it may be well to remember that no community, American or foreign, can point with absolute pride to its history in the care of the defective classes. The proudest and wealthiest

States have reason to bow with shame for errors and failures of duty to the insane. With all the glorious progress of the century just closing, no State can yet feel justified in claiming to have fully discharged its obligations to its dependents and defectives.

The history of the care of the insane in our section of the country shows a gradual process of evolution which may be studied advantageously, since it explains many questions and problems that separately are not easy to be comprehended.

For convenience, an attempt will be made to divide the subject into periods, although, of course, no separate line of demarcation can be drawn as applicable to the whole section. For while one State has made rapid advances, its neighbor may have remained stationary through the indifference or inertia of its people.

FIRST PERIOD.—In colonial days the methods were necessarily primitive. Ideas of demoniacal possession held sway. The treatment was little less than barbarous. The insane were chained in strongly-constructed houses. Paupers were supported by assessment upon the parish in which they lived. In some colonies the laws recognized and made provision for lunatic slaves. The prevailing conception was to protect the sane, and the insane were therefore neglected.

SECOND PERIOD.—About the time of the Revolutionary War, evidence of a better spirit appeared, in that the insane were placed in almshouses, thus passing further under the jurisdiction of the commissioners of the poor. Laymen had official charge of both paupers and lunatics.

In the larger communities, especially in cities, the insane were sometimes assigned to wards and outbuildings of general hospitals. The common designation of these receptacles was "madhouses," and their inmates were held in great contempt. During this period probably little was done in the way of medical treatment, and the lunatics were under charge of brutal keepers.

In some of the States in the early part of the century the insane were also boarded out in private families at a nominal rate determined by the judges of the lower courts and paid by the commissioners of the poor.

THIRD PERIOD.—In the third decade of the century lunatic asylums were founded in Kentucky and South Carolina, and a second institution in Virginia and the Maryland Hospital reorganized. The purpose was partly humane and partly economic. The usefulness of the asylum at Williamsburg had long been demonstrated and its fame spread abroad. The writings and teachings of Dr. Benjamin Rush were also a factor. One or more physicians were naturally found among the leading spirits for establishing asylums. These institutions were built in large towns, and were massive structures. They admitted not only lunatics, but idiots and epileptics. Only the most violent were committed. The asylums were under charge of lay superintendents; in some instances of not a high order of intelligence. Physicians paid visits as the superintendents thought it necessary. The prevailing ideas were altogether custodial. Restraint was common, and violent methods of repression were in vogue, such as shower-baths, tranquilizing chairs, bleeding and vomiting.

The first asylums were planned on a very small scale, and the provisions made for their support exceedingly meager. The rigid adherence of the Southern legislatures to the doctrine that the State should do for the individual nothing which he could do for himself led the legislatures to aim at making asylums self-supporting; that is, the State erected the buildings but the county commissioners of the poor were expected to pay for the support of their paupers.

Payment was required of all who were able to pay. The result of this effort was very unsatisfactory, but it was not abandoned for many years. The official positions were not sought for.

FOURTH PERIOD.—During the decade following 1830 both Tennessee and Georgia established asylums. About 1840 lay superintendents began to give place to "resident physicians" or "medical superintendents," although in some places visiting physicians were continued. Restraint was modified. County care still prevailed, and the profits from pay patients were applied to reducing the expenses of the paupers. Curative treatment began, although the custodial idea was not abandoned. The number of patients even in populous States was small because the prejudice to asylums still prevailed, and county officers encouraged the admission of the violent only. The resident physician was often purchasing and disbursing agent as well as medical adviser. In this period asylums were established in Louisiana and North Carolina.

FIFTH PERIOD.—The decade following 1850 was one of great activity in asylum construction at the South. At the beginning of this period American asylums were said to lead the world. State hospitals were established in Missouri, Mississippi, Texas and Alabama and a second asylum in Kentucky. Dr. Kirkbride's influence was manifest in the plans of construction, and Drs. Galt and Stribling of Virginia, were the recognized leaders in treatment, management and discipline. County care was still continued. The number of patients was so small that no superintendent required more than one assistant physician. A few insane negroes were under care in Maryland, Virginia, South Carolina and Louisiana. The only Southern States not provided with asylums in 1860 were Florida and Arkansas.

The ordeal that asylums passed through during the late war and the period of reconstruction can be realized but faintly now. The demand for soldiers called every able-bodied man to the front, which made serviceable male attendants hard to secure. Clothing was scarce, and worse than all, the food supply was so reduced that often real want stared the hospitals in the face; but through the wondrous providence of God, and the untiring efforts of the self-sacrificing officers, the patients were fed, clothed and sheltered. Upon the close of the war the overthrow of State governments added to the disorder. Another danger menaced the asylums in some of the Southern States in that political interference appeared and proved hurtful to the institutions. It is but due, however, to some State governments during the reconstruction days, to say that they had hearts to sympathize with this afflicted class, and recognized the fact that politics should not interfere with the administration of hospitals for the insane. But the times were hard, State credit was low and everything was uncertain. The administrative officers lived anxious and laborious days; but they stood bravely to their posts and did what they could for the care and welfare of their respective charges, and at last came safely out of the storm. We cannot overestimate the credit due those noble, humane men for their inflexible fidelity to their trust during the time of this turmoil. I have no knowledge of any insane hospital, save one, being closed either during the war or the reconstruction period.

In one particular alone does lunacy administration at the South differ from the same problem elsewhere in our country. What the race problem is to our whole section, so is the question of the colored insane to our specialty. Provision for this class has always been a separate and peculiar problem. Before the war there were, comparatively speaking, few negro lunatics. Following their sudden emancipation their number of insane began to multiply, and, as accumulating statistics show, the number is now alarmingly large and on the increase. This is not the place to enter into an inquiry as to the etiology, but only to recognize the fact and show how earnestly State administrations are striving to meet it.

Those authorities who have given not only most thought to the subject, but who have also dealt with it practically in our asylums, have been unanimous in the opinion that the separation of white and colored patients is to the advantage of both races. The distinction has been made for social reasons alone. Consequently, we find to-day in most Southern asylums four departments, whereas in other institutions two suffice. Virginia and North Carolina have entirely separate hospitals in the center of the negro population near the Atlantic seaboard. This policy has not been deemed advisable in other States, partly for economical reasons but largely because the negro population is more uniformly distributed.

Even the existence of insanity in negro slaves has been questioned. But insanity was common enough to require special provision for the care of lunatic slaves by the Provincial Council of South Carolina in 1745, and the pressing need of means for their accommodation is shown by the early records of our asylums as they were successfully established before 1860.

Prior to the Civil War, in the asylums of Virginia, Kentucky, South Carolina, Maryland, Louisiana and the District of Columbia, colored lunatics were received as patients. In those days the accommodations for negroes were probably not adequate, but a generation ago our predecessors began the work, which we of a later day have in some States not yet been able to carry to the consummation so devoutly to be wished for.

Following their emancipation the negroes have become subject to the same penalties that other races have paid for liberty, license and intemperance. Among those penalties insanity is not the least. A recent estimate, based upon the records at the census office, shows that brain disease in the negro as compared with the whites has increased from one-fifth as common in 1850 and 1860 to one-third as common in 1870 and one-half as common in 1880 and 1890. Or, stated in another way, the ratio of insanity per million among the negroes has risen from 169 in 1860 to 886 in 1890.

Until a recent period, the Southern negro was in a great measure exempt from both insanity and tuberculosis. To-day, associated with insanity, we find tuberculosis alarmingly prevalent among our colored patients, especially in the females. As a race, their mortality is greater than the whites. Medication is of little effect. The tendency of the disease is towards a rapid and fatal decline. If we cannot cure, possibly we may prevent. To this end isolation of tuberculous cases is the most rational method at our command.

I am glad to say from a general survey of the work in the Southern States, that in no section of the country is there a more intelligent and earnest effort made for the insane than there is now made in the South.

All honor to the memory of those who were prominent in the early care and treatment of the Southern insane! The moral grandeur and blessed results of their work can never be estimated. We have taken up their unfinished work and made radical changes and improvements, which have been continuous and progressive, and which have added greatly to the comfort and welfare of the insane.

In my judgment, one of the most rational and humane changes made in the care and treatment of the insane is the abandonment of mechanical and chemical restraint as a general thing. Improved accommodations can be seen in every section of the South. Among other advances may be mentioned the extension of greater liberty to the patients; hospital buildings or wards where the feeble and sick have special care and attention; associated dormitories; better night supervision; training-schools which supply the hospitals with educated nurses, colonies, congregate dining-rooms, amusement halls, chapels, libraries, reading- and working-rooms for convalescents, detached buildings on the cottage plan with homelike surroundings, and every possible means to divert and lead the mind into normal channels; careful and systematic employment, indoors and out, shop and field.

There have been in the past two decades many advances in the clinical, pathological and therapeutic methods, as well as in construction and administration.

It was not until about 1890 that the Southern hospitals may be said to have really recovered from the vicissitudes following the periods of war and reconstruction. The question of bare maintenance and provision for a rapidly increasing population had been paramount. The buildings erected in the last generation have been absolutely free from architectural effect. There has been such need of economy that even to-day many asylums have not a sufficient nursing staff. For a generation it was indeed a struggle for existence.

The study of my subject has taught me that the best and most far-sighted alienists have not proven themselves good prophets when they have undertaken to dogmatize or prophesy about the increase of insanity or about the future policy of States in the management of the insane.

It appears to me that about 1890 our institutions entered upon an era which may be termed the beginning of the scientific period. In the State hospitals generally infirmary wards were introduced and training-schools for nurses were established. These adjuncts are the first essentials of hospital life. Several of our institutions have well equipped, well managed pathological laboratories under skilled neuro-pathologists. From these we expect scientific progress.

The separation of the acute and chronic insane into different institutions has not so far been adopted into the policy of any Southern State. The further segregation of the colored insane will in time, no doubt, become a more important problem. Meanwhile, most Southern States have yet to solve the question of establishing schools for the feeble-minded, asylums for insane criminals, colonies for epileptics and hospitals for inebriates. Although all these questions have been discussed in many States, they have not yet secured following sufficient to obtain legislative aid for their consummation. And yet, before our asylums can be regarded as firmly established on a scientific basis, all these classes must be separated from the insane proper.

Until recently, perhaps too much attention has been paid by neuro-pathologists to the study of nerve tissues to the exclusion of those of the glandular structures of the body.

While such work has been of great value, we are beginning now better to understand that insanity is largely only a symptom, and that many forms of mental derangement present no pathological changes capable so far of demonstration. The intoxication of the cellular nerve elements by auto-infection has of late received much close study and is still being investigated by many of our best workers, as is evidenced by the program of this meeting.

The kidneys of the insane seem to be affected to a much greater degree than are those of persons dying sane. The results obtained in laboratory investigation upon this subject by Dr. Bondurant of the Alabama-Bryce Hospital, Dr. Blackburn of the Government Hospital, Dr. Richardson of Mount Hope, and Dr. Oertel of the Georgia Lunatic Asylum, as well as others, demonstrate the vast importance of careful urinalysis in all cases of mental and nervous disease, and inspire the hope that where timely diagnosis of renal disease is established, the patient may be cured of this condition and at the same time of his mental trouble.

The advances of the past few decades have been such that we are warranted in sustaining the hope that it is within our power to penetrate the mysteries which still enshroud so many vital questions and come at last to a perfect understanding of this wonderful complex nervous system and its subtle life force.

The blood is now receiving the close attention of hundreds of eager trained observers. We have learned the roll of the leucocyte, which for a long time escaped notice altogether, and now differentiate at least five varieties of this cell, some writers describing still other forms.

It is to be hoped that the day is not far distant when every hospital for the insane in this broad land shall have its laboratory in which may be carried out clinical and post-mortem studies which are of so much interest and value to us all.

